# Editorial: Reconstructing social space: spatial dynamics through gendered, cultural and emotional lenses

**DOI:** 10.3389/fsoc.2023.1302905

**Published:** 2023-10-31

**Authors:** Alexandra Macht, Oksana Shmulyar Gréen, Marc Grau-Grau

**Affiliations:** ^1^Department of Sociology, Uppsala University, Uppsala, Sweden; ^2^Department of Sociology and Work Science, University of Gothenburg, Gothenburg, Sweden; ^3^Faculty of Education Sciences, International University of Catalonia, Barcelona, Spain

**Keywords:** space, emotions, gender, migration, care, inequality

How are social spaces experienced and understood? Moreover, what leads to the (re)construction of such spaces through gendered, legal, cultural, and emotional lenses of experience? These initial, intriguing, and broad questions have served as the compass to our Research Topic. We established our commitment to exploring spatial dynamics before transformative events such as the COVID-19 pandemic, the proliferation of virtual interactions, and the war in Ukraine emerged. However, these events have only reinforced the urgency to rethink our social spaces and their dynamics continuously.

We initiated this endeavor with an inclusive and far-reaching mindset, welcoming theoretical and empirical papers, encompassing qualitative and quantitative research. Our primary objective was to assess the research direction that this topic would attract before providing an overview of how the findings addressed the concerns of this Research Topic. After careful consideration and approximately 4 years of scholarly exchanges, we curated the collective insights of this Research Topic of four empirical papers. Departing from the groundwork of Gregory and Urry (1985) and the more recent invitation to spatial sociology by Fuller and Löw (2017), this edited Research Topic aimed to advance an understanding of “space” as a socially produced and relationally constituted concept.

Reflecting on the idea of space is essential in sociology because social reality takes place in various types of space, which are ordering our way of experiencing, perceiving, and performing in the social world. Since its inception, sociology has maintained a profound interest in the effects of living in particular social spaces. A classic example is Simmel, whose work in “The Metropolis and Mental Life” (1903) delves into the psychological effects of living in a city (Simmel, 2002). Simmel's analysis highlights the emergence of a blasé attitude, a form of indifference toward others, as an adaptive response to the excessive stimuli generated in the city (Gałkowski and Kazmierczak, 2021). Similarly, Lefebvre argues in his well-known book, The Production of Space, that space is constructed socially and affects social relations (Lefebvre, 1991). This perspective aligns with Foucault's analysis of how institutions such as prisons employ panopticon surveillance to control individuals and their collective actions. This panopticon perspective has been applied to many other fields, including the university system (Prasad, 2013). Using a Marxist approach, Harvey (1985) also aims to understand how capitalism shapes the production of spaces and organizes neighborhoods according to social classes. In response, Soja (2010) advocates for seeking spatial justice, addressing disparities from these spatial inequalities. Migration, expulsions, and displacements are other contemporary forms of spatial disparities. Sassen explores how “expulsions” occur in the global economy (Sassen, 2014). Not only does the global economy affect physical spaces, but also virtual ones. Castells and his concept of “network society” study how Information and Communications Technology (ICT) reshapes social, economic, and special relations, even the way people date and love (Castells, 2011). So, the advancements in communication and technology have reshaped our experience of time and space by compressing time and shrinking the geographical distance (Giddens, 1990).

In the present day, we can identify many social spaces that shape our experiences, especially the significant intimate spaces of everyday life where we live, care, and work. Even children's rights, which are universal and abstract, are embodied and fully realized (or not) in a particular social space (Grau-Grau et al., 2021). Migration and mobility also create transnational spaces, within which the movement of some people is promoted and for others limited by borders and fences; there are also imaginative spaces, those that live in our memories, or places of social escape like prisons, health wards, or retreats, among others. At the same time, relationships and emotions can create “safe havens” or “living hells,” social media is also a new space for relating and communicating.

Thus, as editors, we sought to include contributions to this field of knowledge that explores how “space” can shape and is shaped through people's everyday lives in different ways. Moreover, the socio-spatial lens can help us to uncover that “spaces are relationally constituted, contestable and processual” (Fuller and Löw, 2017, p. 476). We initially welcomed theoretical and empirical papers on the following suggested or related topics: organizational factors affecting new spaces; household dynamics and new spaces; problematizing the boundaries between private and public spaces; migration and transnational spaces; our memories and imaginative spaces; quality of relations and emotional spaces; social media and non-physical spaces; theoretical approaches to social spaces; how collective issues (ex. a pandemic) influence social spaces. These were some of our initial ideas on the types of Research Topics that aimed at reconceptualizing the concept of “space” and highlighting the empirical findings, drawing on different spatial aspects of human lives.

In its final form, the present Research Topic contains several approaches from international researchers who are (re)conceptualizing the notion of “space” by paying attention to experiences of care and aging, cross-border migration, illiteracy rates, and how emotions shape social distance in two different cultures. The contributors to this Research Topic of peer-reviewed articles were encouraged to pay nuanced attention to institutional or professional, intimate or private spaces inhabited by social actors in their everyday lives. Our initial focus was broad, and we allowed for empirical interpretations of spaces that can become fluid or attain new boundaries through social actors' interactions or how different spatial areas overlapped or merged, if at all. The cultural as well as emotions-focused components of such investigations were, from the beginning, essential factors in the selection of the work we wanted to include, and we are pleased to say that we gathered a diversity of empirical cases conducted within different cultural settings, including research from Switzerland, Germany, China, Romania, and Finland. In the following Research Topic, you can explore how the research presented underlines the links between emotions and gendered attitudes to strangers, opportunity for mobility and the legal status of migrants, education and persistent inequalities, as well as care poverty and its relation to social and spatial emplacement. By including these particular contributions, we highlighted how analyses of the concept of space are not just limited to public, material, or physical elements but how they can also reflect the bodily, emotional, and social/cultural constitutions of different spaces.

In the first article published, Zhang et al. explored, with the help of two studies, the links between anger and its effects on social trust and how this effect might shape the social distance taken between social actors in their interactions with strangers. They used heuristic processing in a quantitative investigation with a sample of 215 German students and a sample of 310 Chinese students, in which they accounted for gendered and cultural variations. The comparative results seem to suggest that gender has more influence on the perception of social distancing than cultural components and specifically that women are more likely than men to exhibit a change in their levels of trust following an angry event. This, in turn, seemed to have influenced women reporting social distancing behaviors. The authors infer that emotions could play an essential part in gendered understandings and enactments of social spacing. The findings are quite unique and deserve an in-depth, qualitative exploration in future research, especially concerning how emotions organize and structure relationships in specific social spaces.

In the second article, Sihto and Van Aerschot show how care and lack of care transformed interviewees' emotional connections with the home space. With the help of 12 semi-structured interviews with customers of outreach work for older adults in three Finnish cities, the researchers hypothesize that lack of care can transform the home into an unsafe space. At the same time, feelings of social exclusion and not belonging can make the boundaries of the home feel like a “safe haven” from the perspective of welfare authorities. By focusing on the emotional impressions and subjective accounts of their participants' lived experiences of safety and exclusion, inclusion, and daily irritations, the authors provide us with a thought-provoking qualitative piece of research in which emotions are meaningful to the reconstruction of the idea of space as a personal and shared experience, in the lives of their interviewees.

The third article in the Research Topic is by Consoli et al., who shed light on the experiences of illegalized migrants and their cross-border experiences in the Canton of Geneva in Switzerland. Through a series of interviews with 39 migrants in combination with the descriptive statistics obtained from panel data, the authors used an inductive thematic analysis to explore fascinating insights into the deeper adjustments of migrants and their subjectivities, identities, and imagined futures. One aspect that makes this research stand out is the discussion of how perceived sources of strength could affect emotional wellbeing. The findings show that having their experiences of cross-border mobility deemed illegal can have relatively lasting effects on social agents and that most of the transformative effects recounted took time to develop.

In the fourth and final Research Topic article, Buza explored the topic of illiteracy and how it was mapped across certain regions in Romania, paying close attention to the differences between rural and urban areas as spatial territories for educational opportunities. By employing census data and adopting a geographical perspective, Buza denotes the peripheralization of illiteracy even though the Romanian educational system adopted Western reforms meant to rehabilitate and streamline the process of helping children from impoverished backgrounds gain access to schools to learn how to read and write. An essential aspect of her careful quantitative research uncovers how ethnic and religious elements contribute to the maintenance of the urban/rural divide with relatively unchanged levels of illiteracy in Romania, especially in the rural environments of the south of the Dobrogea Plain and the Transylvanian Depression region.

Building upon the initial invitation of Gregory and Urry (1985) to reflect on how space and time can be built into the examination of social relations and based on the findings of the papers included in this edited Research Topic, we propose that an expanded definition of space includes internalized, intimate and emotional components, which have previously been neglected in social investigations of space. In their work, Fuller and Löw (2017) theorize the gains of relational spatial research by exploring bodies, borders, units, and mobilities. We expand upon this work by showing how an understanding of space is affected by emotions and gendered subjectivities and is formed in relation to experiences of care, lack of access to education, how legal or socially validated certain forms of identity are shaped, and how emotions are expressed and perceived in the public space. Especially negative emotions of anger or social exclusion have the potential to shape social actors' understanding of how welcomed they feel within a given social space and how safe they perceive their social environment to be; according to whether they feel anger or not, an individual's social space could be expanded through experiences of trust and social cohesion or shrunk by various forms of anger and not being welcomed (or as Buza has shown, not being educated enough). As interesting as the findings of the 4 papers of this Research Topic are, they are an incentive to open up discussions rather than lock them into a comforting sense of completion. This is because, with our edited Research Topic, we aim to keep reconceptualizing the idea of social space rather than have the final word on its definition.

What is nonetheless comforting is seeing now how the Research Topic formed finally into a solid whole, seeing as our working efforts were marred in the last years by a global pandemic and the armed conflagration between Ukraine and Russia, which affected the personal lives of two of the members in our editorial team. Finally, we hope the findings included in this edited Research Topic will provide some food for thought in upcoming scholarly debates. We are thankful to the Frontiers team for providing us with a platform to showcase these critical studies, and we look forward to fruitful future collaborations.

## Author contributions

AM: Conceptualization, Project administration, Supervision, Writing—original draft, Writing—review & editing. OSG: Conceptualization, Project administration, Supervision, Validation, Writing—review & editing. MG-G: Conceptualization, Writing—review & editing.
